# Exploration of identifying individual tumor tissue based on probabilistic model

**DOI:** 10.3389/fonc.2024.1297135

**Published:** 2024-04-23

**Authors:** Yuhan Hu, Qiang Zhu, Xuan Dai, Mengni Zhang, Nanxiao Chen, Haoyu Wang, Yuting Wang, Yueyan Cao, Yufang Wang, Ji Zhang

**Affiliations:** ^1^ Department of Forensic Genetics, West China School of Basic Medical Sciences and Forensic Medicine, Sichuan University, Chengdu, China; ^2^ Department of Pathology, West China Hospital, Sichuan University, Chengdu, China; ^3^ College of Computer Science, Sichuan University, Chengdu, China

**Keywords:** probabilistic model, likelihood ratio (LR), tumor source identification, short tandem repeat (STR), forensic genetics

## Abstract

Variations in the tumor genome can result in allelic changes compared to the reference profile of its homogenous body source on genetic markers. This brings a challenge to source identification of tumor samples, such as clinically collected pathological paraffin-embedded tissue and sections. In this study, a probabilistic model was developed for calculating likelihood ratio (*LR*) to tackle this issue, which utilizes short tandem repeat (STR) genotyping data. The core of the model is to consider tumor tissue as a mixture of normal and tumor cells and introduce the incidence of STR variants (*φ*) and the percentage of normal cells (*M_xn_*) as *a priori* parameters when performing calculations. The relationship between *LR* values and *φ* or *M_xn_* was also investigated. Analysis of tumor samples and reference blood samples from 17 colorectal cancer patients showed that all samples had *Log*_10_(*LR*) values greater than 10^14^. In the non-contributor test, 99.9% of the quartiles had *Log*_10_(*LR*) values less than 0. When the defense’s hypothesis took into account the possibility that the tumor samples came from the patient’s relatives, *LR* greater than 0 was still obtained. Furthermore, this study revealed that *LR* values increased with decreasing *φ* and increasing *M_xn_*. Finally, *LR* interval value was provided for each tumor sample by considering the confidence interval of *M_xn_*. The probabilistic model proposed in this paper could deal with the possibility of tumor allele variability and offers an evaluation of the strength of evidence for determining tumor origin in clinical practice and forensic identification.

## 1 Introduction

Tumor tissue, especially formalin-fixed and paraffin-embedded (FFPE) samples, may be the only source of biological material available for individual identification or kinship analysis (1–6). During tumorigenesis, variations are constantly occurring and accumulating in the cell genome (7). Variants, such as deletions and increases of alleles associated with multiple genetic markers, have been observed in tumor tissues. These result in inconsistent genotyping results compared to normal tissue or blood samples from the same individual. In addition, the allele frequencies used in identification statistical analysis typically do not take into account the effects of disease states such as tumors. This poses a significant obstacle in tumor source identification.

Several retrospective studies have examined the variation pattern and rate of short tandem repeat (STR), a highly polymorphic, easily detectable, and commonly utilized genetic marker (8), and selected specific loci with low variation rates for individual identification of tumor tissue (9–12). Poetsch et al. classified the STR variants observed in tumor tissue into three categories as follows: 1. the additional alleles (Aadd), 2. the new alleles instead of somatic-derived alleles (Anew), and 3. partial or complete loss of heterozygosity (pLOH/LOH), and provided criteria to distinguish LOH from pLOH (13). The possibility of using nuclear genomic SNPs (14, 15) and indels (16), along with mitochondrial SNPs (5, 17) has also been investigated for tumor source identification. However, the aforementioned retrospective study analyzed the existing experimental data empirically. It exclusively selected non-mutated loci for individual identification. Nevertheless, this approach was constrained by the sample size and lacked a statistical metric to quantify the strength of the evidence, i.e., the genotyping profile makes the tumor sample originating from the person of interest more or less probable. Furthermore, the genetic marker indel, which is more significant in mutations, cannot be used to identify the body origin of tumor tissue (16). This is also the case for mitochondrial SNPs due to their heterogeneity.

In research focused on statistical methodologies for discerning the individual origin of tumors, one strategy is to consider tumors as a mixture of tumor cells and normal cells, and treat the two components as independent individuals with a certain level of “genetic relationship.” Based on this assumption, identity by state (IBS) analysis was applied to perform body source identification of tumor samples (18–20). However, these studies did not consider the causes and patterns of STR variation in tumor cells and only provided a conclusion that “cannot be excluded.” Additionally, this approach was insufficient for assessing the strength of evidence for DNA analysis of tumor samples.

It has been shown that the DNA of tumor tissue exhibits a mixed composition, which contains the normal cell population and abnormal cellular subclones arising from the branching evolutionary growth pattern of the tumor (21). Alterations in chromosome number and structure, as well as numerous changes at the genomic level, can be observed in these abnormal cells (7, 22) so that the different STR variants described above were observed when these variations were reflected on the STR profiles. In this case, the frequency of the tumor-derived alleles is no longer equal to the generally used population allele frequency, and this change is associated with the incidence of STR variants. The goal of this study was to develop a probabilistic model for tumor source identification that incorporates the incidence of STR variants and provides a measure of the evidence strength.

The likelihood ratio (*LR*) provides a numerical value that indicates the relative strength of the evidence for the prosecution’s hypothesis (typically that the evidence came from the person of interest) compared to the defense’s hypothesis (that the evidence is from an unrelated source) (23). In the present study, we attempted to apply a probabilistic modeling strategy for traditional STR profile to tackle the challenge of genomic variation in tumor identification and provide metrics for evaluating the strength of evidence. We innovatively introduce the incidence of STR variants (*φ*) and the percentage of normal cells (*M_xn_*) as the *a priori* parameters into the tumor source identification pipeline, which leads to a reasonable and effective *LR* calculation.

## 2 Materials and methods

### 2.1 Sample collection

Colorectal tumor tissue from 17 patients were collected and fixed in 10% neutral-buffered formalin solution for 48–72 h. The tumor samples were treated routinely with xylene transparency following progressive ethanol dehydration, then paraffin embedding to make FFPE samples. Hematoxylin- and eosin-stained slides were reviewed. A pathological diagnosis and the percentage of tumor cells *M_xt_* (24) for each slide were provided. The percentage represented the proportion of tumor cells to the total cell area observed under the microscope. Determination was reached through a consensus of two pathologists with over a decade of experience in molecular pathology (25). Based on this, the percentage of normal cells *M_xn_* was calculated as 1-*M_xt_*. Peripheral blood from the corresponding patients was collected as reference samples. The tumor samples were designated as “HTFD”, while the blood samples were labeled as “HBD”.

### 2.2 DNA extraction, PCR amplification, and STR typing

Five 10-µm serial sections were obtained for all FFPE samples with the first two to three sections discarded. DNA extraction for both FFPE sections and blood samples was performed using the QIAamp^®^ DNA Mini Kit (QIAGEN, Germany) according to the manufacturer’s instructions. All samples were quantified fluorescently using the Qubit^®^ dsDNA HS Assay kit (Invitrogen, USA). A total of 34 DNA samples were diluted to 1 ng/μl with nuclease-free water as templates. STR-targeted amplification was performed in a total volume of 5 μl using the GlobalFiler™ PCR Amplification Kit (Applied Biosystems, USA). The standard protocol of 29 cycles was used on a Veriti™ 96-Well thermal cycler (Applied Biosystems, USA). Negative controls were always included in the same batch for amplification.

Then, 1 μl of PCR products was mixed with 8.9 μl of Hi-Di formamide (Applied Biosystems, USA) and 0.1 μl of SIZ-500 (AGCU, China) DNA-size standard. Amplicon separation and peak height signal acquisition were carried out on a 3500 Genetic Analyzer (Applied Biosystems, USA) using a 36-cm capillary and POP-4 polymer (Applied Biosystems, USA) with an injection voltage of 1.2 kV and an injection time of 5 s. Data analysis was conducted using GeneMapper ID-X 1.5 software (Applied Biosystems, USA). The AT value was 175 RFU, and locus-specific stutter filtering was performed according to the manufacturer’s instructions, while all other analysis methods were set by default. The data were exported in text format and contained details regarding allele typing and peak height.

To ensure reproducible and credible results, STR typing would be repeated if the genotyping results between the reference sample and the tumor tissue are inconsistent.

### 2.3 Theoretical considerations

DNA profiles generated from tumor tissue were always DNA mixture profiles because solid tumor tissue is composed of not only tumor cells but also tumor-associated normal epithelial and stromal cells, immune cells, and vascular cells (26). The following mutually exclusive assumptions was made to assess the strength of the evidence that the tumor tissue came from a potentially known individual:

*H_p_*.: The tumor tissue is composed of normal cells and their tumor cells from a known individual.
*H_d_* : The tumor tissue is composed of normal cells and their tumor cells from a random unrelated individual.

The *LR* was determined by:


$$LR\;=\;\frac{P\left(E|H_{p}\right)}{P\left(E|H_{d}\right)}$$

#### 2.3.1 Basic assumption

**Assumption 1.** The STR-CE peak height data generated from tumor samples, which was the object of modeling in this research, also follow the gamma distribution.

During the PCR procedure for DNA, the copy number of the targeted fragment increased with the number of cycles in a binomial distribution (27). Since the peak height detected by the Genetic Analyzer is a measure of the copy number of the PCR end product, it is also subject to stochastic effects throughout the PCR process. Therefore, the peak height data generated from the tumor tissue complies with this principle and follow the gamma distribution (28).


Y ~ɡ amma(1ω2,μω2)

where *μ* is the peak height expectation, and *ω* is the coefficient of variation of the peak height (29). The sum of peak heights for each autosomal locus of individual plots was fitted to gamma using maximum likelihood estimation, and quantile–quantile (Q-Q) plots were drawn to confirm the fit of the data to the gamma distribution.

**Assumption 2.** The height contributions of different cell populations are independent.

Tumor cells are derived from normal cells by mutation, which could generate endogenous mitogenic signals resulting in independent proliferation (30). As a result of ongoing genetic mutations that occurred in tumor cell populations derived from a founder cell, intratumor heterogeneity and different subclones, each of which is a rather stable, homologous cell population with identical genetic composition and independent of each other, are produced according to the clonal evolution model (21).

**Assumption 3**. STR locus *M* was independent of each other and in a state of linkage disequilibrium (31).

**Assumption 4**. Alleles of the specific locus $A_{m}=\left\{a_{1},a_{2},\ldots ,a_{i}\right\}\;$ are independent of each other.

The allele frequencies in this research were derived from a population survey of Southwest Han Chinese individuals (32). Therefore, given the assumptions *H* and the parameters *μ ω*, the probability of observing the profile *E* can be written as:


p(E∣H)= ∏m=1M∑ɡ m∈ Gm p(ɡ m∣H)∗p(ym∣ɡm,μ,ω)

where $g_{m}$ is the set of genotypes for different cell populations and one of the different genotype combinations $G_{m}$ at locus *m*.

#### 2.3.2 Number of contributors

Usually, normal cells are in genomic concordance and can be considered as a homogeneous cell population. However, there may be two or more subclones of abnormal cells. Due to variations in the tumor genome, an STR genetic marker may be present in these subclones with different alleles. Thus, a tumor tissue sample may have a mixed genotype from two or more different cell populations, i.e., a normal cell population and K−1 tumor subclones, where *K* is the total number of cell populations. In this study, the maximum allele count (MAC) method was used to estimate the minimum number of the cell populations corresponding to the number of contributors in tumor tissue. The formula used to determine the minimum number of contributors was as follows:


$$k_{min}=\{\begin{array}{c} 2,\;L_{max}<3\\ ceiling\frac{L_{max}}{2},\;L_{max}\geq 3 \end{array}$$

where *ceiling x* denoted rounding up by *x* and *L_max_* denotes the maximum number of alleles among loci. Based on the above, the two hypothetical propositions could be expressed as follows:

*H_p_* : The tumor tissue is composed of normal cells and their *K*–1 tumor subclonal cell populations from known individuals.
*H_d_* : The tumor tissue is composed of normal cells and their *K*–1 tumor subclonal cell populations from random, unrelated individual.

#### 2.3.3 STR variation model

**Assumption 5.** Genotypes among cell populations are independent given the incidence of STR variants (*φ*).

In previous research, STR variants in tumor tissue showed the emergence of new alleles and the loss of alleles compared to normal reference samples. In the present study, the incidence of STR variants was assumed to be *φ*. When the tumor-derived allele was inconsistent with the allele of normal cell after genotype combination at locus *m*, the frequency of the tumor allele was determined by multiplying the population frequency of the somatic allele by the *φ_m_* value. The calculation formula was as follows:


$$P\left({ɡ}_{m}|H_{\;},\varphi_{m}\right)=P\left({ɡ}_{n}|H_{\;}\right)*\prod_{k=1}^{K-1}P\left({ɡ}_{t,k}|H_{\;},\varphi_{m}\right)$$

where *g_n_* is the genotype of the normal cell population, and *g_t,k_* is the genotype of the *k*-th tumor subclones. The origin of variation in each allele of the *g_t,k_*, as well as all possible scenarios, were considered. More details can be found in **Supplementary Tables 1**–**3**.

The *φ* value varied across different loci within the same type of tumor. In colorectal cancer, the range of *φ* for 19 STR loci is [7.75%, 43.41%] (9). To evaluate the effect of *φ* and *M_xn_* as *a priori* parameters on the calculated values of *LR*, the following two experiments were set up:

Experiment 1. The maximum likelihood values were obtained by taking 21 values each in the confidence interval of [*M_xn_* ± 10%] and the range of *φ* to form a 21 × 21 combination of *a priori* parameter matrix under *H_p_* and *H_d_*, and then *LR* was calculated.

Experiment 2. The maximum likelihood values were obtained by taking 21 values each in the confidence interval of [*M_xn_* ± 10%] while *φ* took the incidence of variants at the corresponding loci. If the locus was not included in the reference (9), the value was substituted with the average value.

#### 2.3.4 Degradation model

Tumor tissue was fixed with formaldehyde during FFPE sample preparation. Formaldehyde-mediated hydroxymethylation of the imino and amino groups of the DNA molecule would result in irreversible denaturation of the DNA molecule and cross-linking of DNA and proteins to prevent protease digestion of the tissue and obstructing nucleic acid extraction. Due to methylene cross-linked bridges between biomolecules, DNA would become more brittle and prone to random breaks when subjected to shear force (33). DNA enzymes also play a role in this process (34). Consequently, the STR profile of FFPE samples showed a “ski-slope-like” profile where the peak height declined with increasing molecular fragment size (35), thus FFPE samples were considered as one of the typical DNA degradation samples in the forensic field (36).

In this study, a degradation model was employed, which was developed based on the research of Tvedebrink et al. (37): the probability of breakage between any two bases in a sequence was uniform. Subsequently, a log-linear model was employed to describe the correlation between the average peak height *H* and the average fragment size *bp* at the locus *M*. After this, the initially estimated degradation coefficients could be obtain using the least squares method. An assessment can then be conducted to determine whether to incorporate degradation parameters in the model. Finally, the parameter of the gamma model could be scaled with the expression $\varepsilon^{\frac{bp_{m,a}-90}{100}}$ for allele a at maker *m* to account for degradation if needed (38).

#### 2.3.5 Other parameters

Because the sample source was tissue cells and the PCR input template amount was 1 ng/μl, the total peak height of each allele in different profiles, including Aadd, was much higher than the analysis threshold. However, most of the drop-in events had lower peak heights. This meant that the probability of high peaks being drop-in events was significantly reduced (39). At the same time, the negative control of the same batch without allelic calling could indicate a minimal probability of drop-in and contamination (40). Stutter was filtered by the appropriate locus threshold according to the kit protocol. Therefore, to avoid making the model more complex, neither the drop-in parameter nor the stutter parameter was introduced in this study. Furthermore, when the value of K exceeded 2, indicating the presence of more than one subclone of tumor cells, it became necessary to estimate the proportion of the tumor cell population ( $M_{xt_{1,2,3\ldots }}$ ), and the variable satisfied the following formula:


$$1-M_{xn}=\;\sum_{k=1}^{K}M_{xt_{k}}$$

Thus, the probability of observing the profile *E* can be written as follows:


$$p\left(E|H\right)=\;\prod_{m=1}^{M}\sum_{g_{m}\in \;G_{m}}^{\;}p\left({ɡ}_{m}|H,\varphi_{m}\right)*p\left(y_{m}|{ɡ}_{m},M_{x},\mu ,\omega ,\varepsilon \right)$$

where *φ_m_* and *M_xn_* are *a priori* parameters that are derived without optimization parameter search.

### 2.4 Hd true test

The non-contributor test was performed to verify the specificity of the *LR* results (41). The profiles of 1,000 unrelated individuals were randomly generated according to the above frequency table (32), which included 21 autosomal STR loci in the Globalfiler™ kit, and then was adopted as person of interest to calculate *LR*. The *a priori* parameter *M_xn_* was divided into five groups, which were the maximum, minimum, and quartile values in the range of the *M_xn_* interval, and each group included 200 unrelated individuals for the non-contributor test.

### 2.5 Relatedness consideration

As mentioned above, there is a kinship-like genetic relationship between normal cells and tumor cells. To validate whether the model can distinguish the origin of tumor tissue between a true contributor or a close relative of the contributor, this study also performed the analysis of simulated related individuals, including the parent–offspring (PO) and the full-sibling (FS) relationships, while keeping the rest of the model constant.

Here, the following two *H_d_* propositions have been implemented:

*H_dPO_* : The tumor tissue is composed of normal cells and their *K*-1 tumor subclonal cell populations from a known individual’s PO.
*H_dFS_* : The tumor tissue is composed of normal cells and their *K*-1 tumor subclonal cell populations from a known individual’s FS.

The above pipeline was implemented using a Python script that is publicly available on GitHub (https://github.com/HYH-yuhan/TumorID). Through this pipeline, *LR* could be calculated for each tumor tissue STR profile and the corresponding reference.

## 3 Result

### 3.1 Sample overview

The pathological type of all tumor samples was adenocarcinoma. As shown in **Table 1**, the percentage of tumor cells under HE staining was >30% in each of them. The highest incidence of Aadd was observed among the three mutation types that would result in STR genotype alteration, while no Anew was observed. Specifically, the sample HTFD5719 showed heterozygosity at five loci, whereas its reference HBD5719 was originally homozygous. Five loci displayed three alleles, and two loci exhibited five alleles. The maximum number of alleles for the remaining samples with Aadd was three.

**Table 1:** Microscopic details and STR profile information of all 17 tumor tissue samples.

Sample name	M_xt_*	Number of alternated loci	Aadd	Anew	LOH	Degradation slope
HTFD 0166	60%	1	1	0	0	0.996152
HTFD 0471	50%	3	1	0	2	0.996633
HTFD 1504	60%	0	0	0	0	0.997766
HTFD 1611	30%	1	1	0	0	0.994265
HTFD 2408	70%	3	1	0	2	0.996342
HTFD 3418	80%	5	1	0	4	0.995502
HTFD 4084	40%	2	2	0	0	0.997634
HTFD 4107	70%	1	1	0	0	0.997137
HTFD 4336	60%	1	1	0	0	0.996315
HTFD 5200	80%	0	0	0	0	0.995292
HTFD 5211	70%	0	0	0	0	0.995772
HTFD 5719	60%	12	12	0	0	0.995084
HTFD 6566	40%	0	0	0	0	0.998483
HTFD 6722	60%	0	0	0	0	0.994964
HTFD 6815	90%	0	0	0	0	0.994535
HTFD 7709	80%	3	0	0	3	0.994679
HTFD 9601	80%	0	0	0	0	0.995879

*Mxt, the percentage of tumor cells, is determined visually from professional pathologists.

### 3.2 Fitting results of peak height and degradation

**Figure 1:**
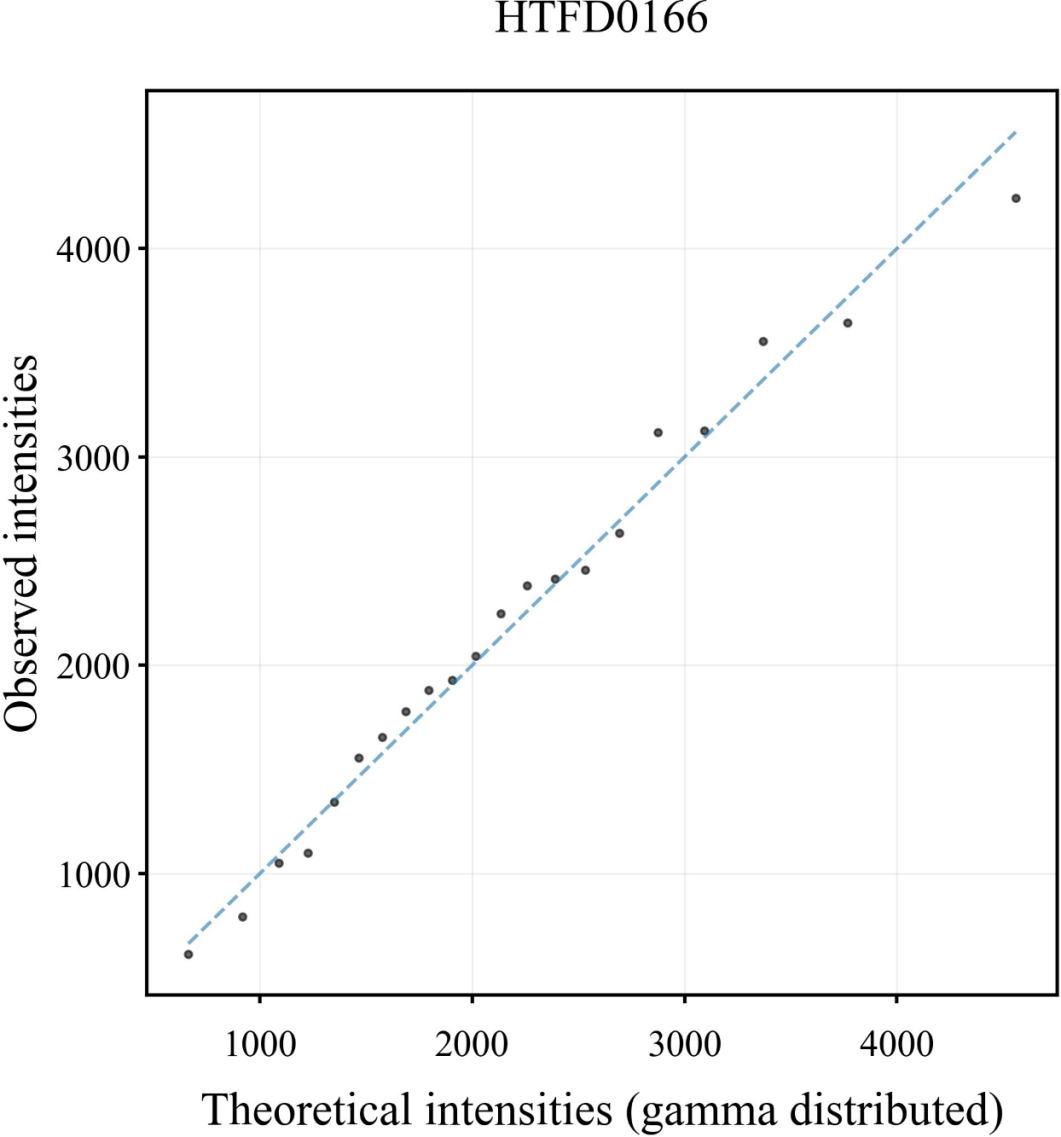
Q-Q plot of peak height derived from the DNA profile of sample HTFD0166.

In the Q-Q plot of the peak height for the sample HTFD0166 (**Figure 1**), the scatter was basically distributed along the y = x line indicating that the observed peak height exhibited a strong correspondence with the gamma distribution. The Q-Q plots of the other samples are shown in **Supplementary Figure 1**, and displayed similar distribution characteristics are as depicted in **Figure 1**.

The degradation slope, representing P(No breakage between a given base pair) , of 17 profiles was initially fitted with a log-linear model. The probability of degradation per base pair, denoted by P(deg) , was equal to ( 1−degradation slope ). The P(deg) for 17 profiles ranged from 0.001517 to 0.005735 according to **Table 1**. As described in (42), the probability of intact fragments available for amplification is approximately 25%–75% at 200 bp. Therefore, the incorporation of the degradation parameter *ε* is needed to be considered in subsequent studies.

### 3.3 *LR* results

#### 3.3.1 Experiment 1

**Figure 2:**
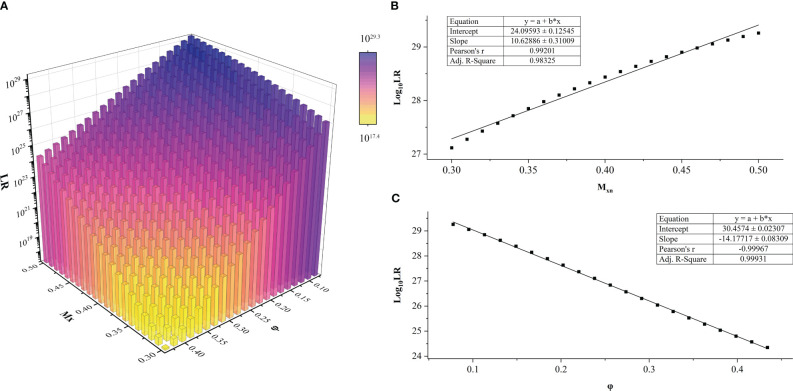
*LR* values of sample HTFD0166 from Experiment 1. **(A)** *LR* results obtained within the parameters of gradient *φ* and *M_xn_*, *LR_max_* = 10^29.3^; *LR_min_* = 10^17.4^. **(B)** When *φ* was fixed, *Log*_10_ (*LR*) varied linearly with *M_xn_*. This graph was illustrated with *φ* = 0.0775. **(C)** When *M_xn_* was fixed, *Log*_10_ (*LR*) varied linearly with *φ*. This graph was illustrated with *M_xn_* = 0.5 as an example.

A total of 441 *LR* values were calculated for each DNA profile of the FFPE sample. The dependence of *LR* values on the *a priori* parameters *M_xn_* and *φ* in HTFD0166 was analyzed (**Figure 2A**). The maximum value of *LR* could be calculated when *M_xn_* was the smallest and *φ* was the largest. Conversely, the minimum value of *LR* could be obtained when *M_xn_* was the largest and *φ* was the smallest. *Log*_10_ (*LR*) increased with increased *M_xn_* or decreased *φ*, and there exists a linear correlation between the variables. A linear fit through R showed that, for example, when φ=0.0775 , $Log_{10}(LR)=(10.62886\pm 0.31009)M_{xn}+(24.09593\pm 0.12545)$ , Pearson’s r was 0.99201, and adjusted *R* ^2^ was 0.98325 (**Figure 2B**). In another situation, when $M_{xn}=0.5$ , $Log_{10}(LR)=(-14.1771\pm 0.08309)\varphi +(30.4574\pm 0.02307)$ , Pearson’s r was −0.99967, and adjusted *R* ^2^ was 0.99931 (**Figure 2C**). Among all 17 profiles, adjusted *R* ^2^ and Pearson’s r for the linear fits of $Log_{10}\left(LR\right)$ to *φ* were between [0.9003, 0.9999] and [0.9513, 0.9999], respectively. For the same fits to *M_xn_*, they were between [0.9674, 1.000] and [−1.000, −0.9844], respectively. Both showed a strong linear correlation (43). For more details, refer to **Supplementary Figure 2** and **Supplementary Tables 4**–**20**.

#### 3.3.2 Experiment 2

**Figure 3:**
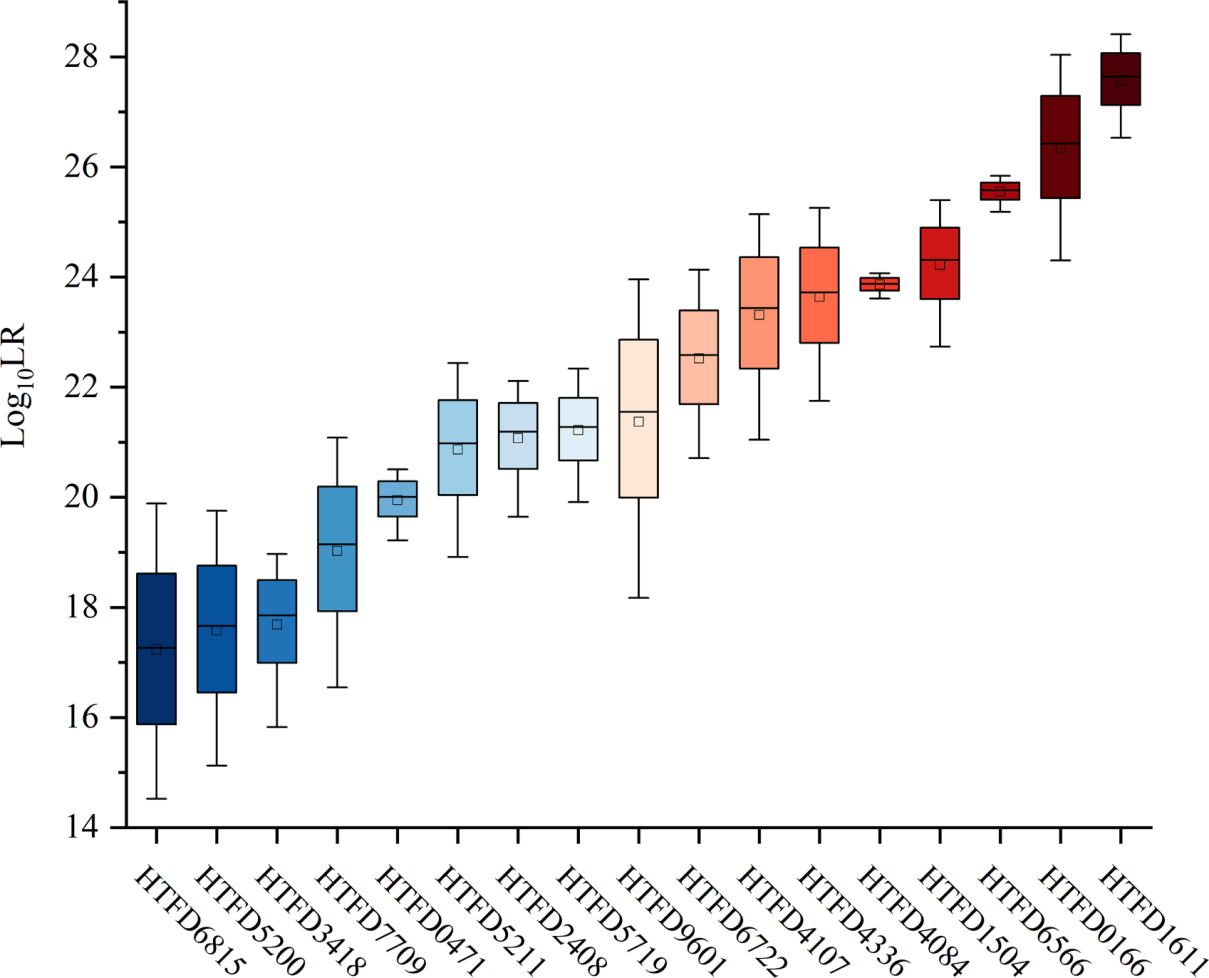
*Log*_10_ (*LR*) values of all 17 tumor tissue samples.

The $Log_{10}\left(LR\right)$ value was linearly increased with *M_xn_* in each of the samples (**Figure 3**). The maximum average of $Log_{10}\left(LR\right)$ was 27.6370 with sample HTFD1611, which had the largest *M_xn_* in **Table 1**; the minimum average of $Log_{10}\left(LR\right)$ was 17.2624 belonging to HTFD6815, which had the smallest *M_xn_*. All $Log_{10}\left(LR\right)$ values were much larger than 1, which significantly supported the prosecution’s hypothesis that the tumor tissue was composed of normal cells and their K−1 tumor subclonal cell populations from known individuals.

### 3.4 Hd true test results

**Figure 4:**
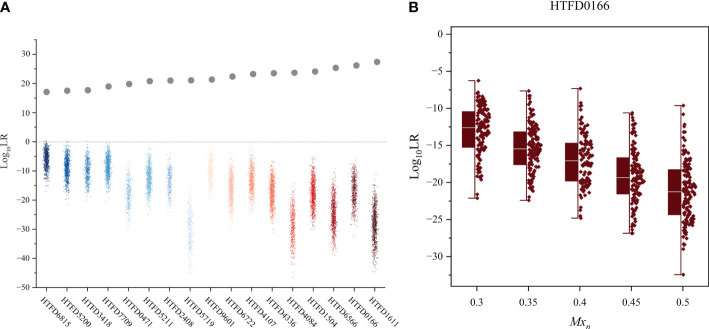
Hd true test results. **(A)** *Log*_10_ (*LR*) value of the non-contributor test for all 17 samples, while the gray dots indicate the mean *Log*_10_ (*LR*) values for the true contributor in Experiment 2. **(B)** Distribution of *Log*_10_ (*LR*) values for the non-contributor test of sample HTFD0166 separated by the percentage of normal cells (*M_xn_*).

The 99.9% of the $Log_{10}\left(LR\right)$ values for the non-contributor tests of all samples was less than 0 (**Figure 4A**), which did not support the prosecution’s hypothesis. In addition, the *LR* values of the non-contributor test tended to decline as *M_xn_* increased, as illustrated in **Figure 4B** for sample HTFD0166. The Hd true test results for all samples are shown in **Supplementary Figure 3**, where the four nonoutlier data points in the box plot were above 0. That is, LR=1.6082/1.7161/1.2141 for sample HTFD6816 when $M_{xn}=0.01$ , and LR=1.0948 for sample HTFD5200 when $M_{xn}=0.1$ . In Experiment 2, these two samples had the lowest $Log_{10}\left(LR\right)$ among all results.

### 3.5 *LR* results considering kinships

**Figure 5:**
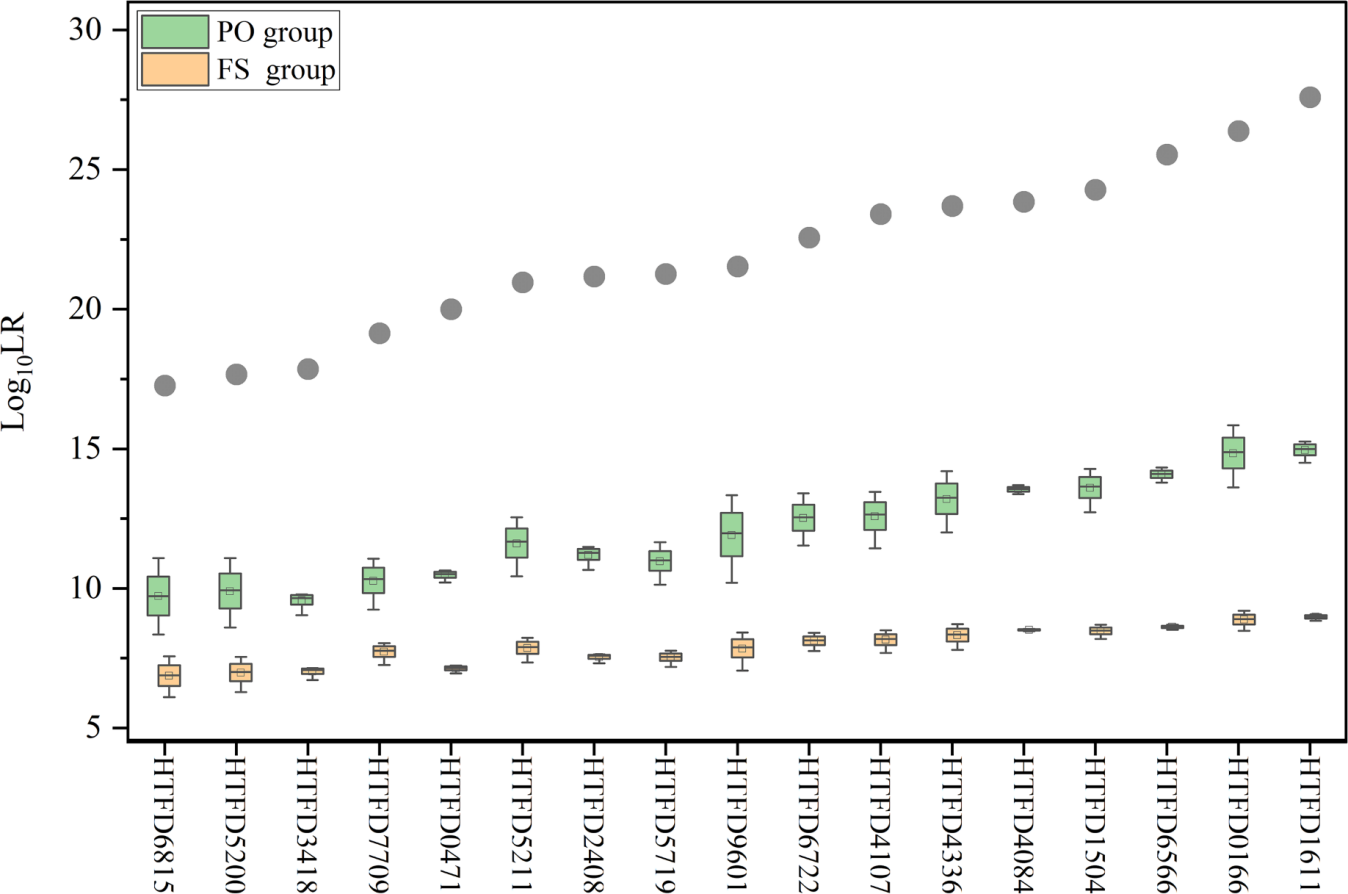
*Log*_10_ (*LR*) value while accounting for kinship between contributors. The gray dots indicate the mean *Log*_10_ (*LR*) values for true contributors in Experiment 2, and the green box plot represents the results of *Log*_10_ (*LR*) for the PO group and the yellow for the FS group.

When relatedness to known individuals was considered under *H_d_*, *LR* became smaller in different degrees, but still greater than 1, which did not support the hypothesis that the individual related to the true contributor was a contributor. The $Log_{10}\left(LR\right)$ calculated for the true contributor > $Log_{10}{\left(LR\right)}_{PO}\;$ > $Log_{10}{\left(LR\right)}_{FS}$ . Meanwhile, as the $Log_{10}\left(LR\right)$ increased $Log_{10}{\left(LR\right)}_{PO}$ and $Log_{10}{\left(LR\right)}_{FS}$ of the corresponding groups showed an increasing trend (**Figure 5**).

## 4 Discussion

In the current study, we constructed a probabilistic method based on the gamma model and an *LR* computational framework that takes into account STR variants in tumor cells. To the best of our knowledge, this is the first study to introduce a mixed DNA and probabilistic approach for tumor source identification. High *LRs* greater than 10^14^ were obtained for all samples distinguishing the true contributor from random unrelated individuals and potential relatives of contributors. In addition, the quantitative strength-of-evidence indicator provided a more scientific solution for tumor source identification. Adopting the confidence interval of *M_xn_* could result in the calculation of a conservative statistic. However, the following issues need to be considered.

### 4.1 Effect of tumor genomic variants on the STR profile

Cancer cells undergo multiple genetic hits during tumorigenesis, including somatic point mutations, copy number variants, gene deletions, gene rearrangements, and translocations (7). These variants are the source of the abnormal STR profiles observed in tumor tissue. First, point mutations in the primer binding region can result in null alleles of the STR. Second, gene deletions or loss of heterozygosity (LOH) can cause somatic loss of wild-type alleles in many hereditary cancer syndromes. Knudson’s two-hit hypothesis for LOH suggests that one copy of the tumor-suppressor gene is inactivated by mutation, and the other copy undergoes mitotic recombination/gene conversion or deletion, the former leading to copy number losses (CNL-LOH) and the latter to copy number neutral losses (CNN-LOH) (44). In addition, the chromosomal region of LOH contains the location of some loci in commercial STR kits (45), which is reflected by the loss of alleles in the STR profile, and the precise mechanism of the allele loss is unknown.

Furthermore, as a type of microsatellite, the tandem repeat structure of STR also has the potential to generate new alleles due to the strand-slippage replication in tumor cells with vigorous growth and defective DNA repair mechanisms (22, 46). This phenomenon is also used to detect microsatellite instability (MSI) to evaluate tumor hypermutability (9).

### 4.2 Reasonableness of the STR variation model

In addition to the diversity of mutations that occur in the genomes of tumor cells, neoplasms arise from a single somatic cell of origin (47) and undergo a clonal evolution to form distinct subclones (21). The genomes of these subclones will possess the same or different variants, but still have regions identical to the genome of the original normal cells. This underlying theory provides a theoretical foundation for using the probabilistic strategy in this study to deal with the variation or invariance of tumor alleles.

As tumors develop, the genome becomes increasingly unstable, and the likelihood of mutations during cell proliferation and differentiation increases significantly with each generation. Thus, the developmental expansion of tumors is a branching clonal structure rather than a linear clonal evolution (21, 48). According to computer simulations, each subclone represents a relatively stable, homologous population of cells (49). Such subclonal characteristics of tumor tissue implies that a tumor cell subclone may be represented by an unknown contributor in the probabilistic model. It also implicates that dynamics of each stage of tumor evolution cannot be accurately predicted, i.e., the probability that a particular allele will be altered and the type of mutation that will occur. Therefore, the cumulative mutations in a cell’s genome as an overall probability was considered to describe the likelihood of allelic variants as well as the potential for all alleles in tumor cells to mutate. That is, the probability of gain or loss of an allele depends on the corresponding STR variant, which is derived from empirical statistics of the incidence of STR variations (*φ*).

Another consideration for using the empirical variant incidence is that new or missing alleles arising from tumor mutations are usually associated with the source allele. Furthermore, the variant rate is a conditional probability based on the assumption that the origin of the tumor-derived alleles is determined, and it serves as a substitute for the frequency of the tumor allele in the population within our model. Given the inability to ascertain the specific subclone in which the variant allele is present, the possibility of each allele being variant at all loci was taken into account.

### 4.3 Consideration of φ and *M_xn_*

Specifically, two types of variations in the STR profile were observed. One is the loss of alleles, which can result from the dropout of normal cells in a trace state or from the loss of heterozygous of tumor cells. Any allele dropout occurring in normal cells was considered for conservativeness because if the sensitivity of the detection platform was insufficient, normal cellular alleles in trace amounts would not be detected (19). Heterozygous loss of every tumor cell alleles at all loci was also taken into account, as the region of LOH has been continuously discovered with advances in detection technology and bioinformatics (50). In this model, the probability of LOH was considered within a given genotype combination. The other situation is the presence of additional alleles, which is the appearance of abnormally long or short microsatellites on the profile. The possibility for simultaneous variation arising from a pair of alleles was taken into account when examining the inconsistency between tumor cell and normal cell genotypes during the process of genotypic permutations. As a result, the result tends to be conservative. In short, the incidence of STR variants (*φ*) was considered for each allele at all loci for conservation.

The genetic alterations in carcinomas are chromosome specific (51), and previous studies have found that these loci had different degrees of mutation. For example, TPOX had a low mutation rate in a wide range of tumors (9–11). Therefore, different STR variation probabilities were assigned to each locus based on the population survey results. However, it has been observed that certain loci exhibited varying rates of mutation across different types of cancer. The mutation rates tend to rise in more aggressive forms of cancer (10) and has been shown to be associated with the population average heterozygosity and variability of the repeat number of microsatellite loci (52). The samples and model parameters utilized in this study were limited to primary colorectal cancer tumors, whereas further research is required to validate the findings on tumors of various types and from diverse population backgrounds.

Estimating the percentage of cancer cells in a solid tumor sample, denoted by “tumor purity,” which is the same as *M_xt_*, has been an active research topic. Published studies have evaluated tumor purity assays using complex and expensive genome-wide (53), exome (54), or transcriptome data (55). Patel et al. reviewed several algorithms for estimating tumor purity. However, none of the computational approaches for estimating tumor purity achieved the status of being universally accepted as the “gold standard.” The most consistent analysis was between two pathologists using light microscopy (24). In this study, the microscopic analysis for tumor cell proportion is utilized for convenience. Given the inherent bias associated with manual estimation, *LR* interval values were calculated within the dynamic range of *M_xn_*. This approach enhances the credibility and validity of the obtained results. In the future, there is potential for the concurrent estimation of tumor purity and individual identification using large-scale sequencing data, which remains to be explored.

It should be noted that we attempt to use the *k*-value to describe copy number variation for simplicity. However, the current *k*-value estimation relies solely on qualitative information and cannot use the peak height influenced by copy number. This results in an underestimation of the *k* value, and further investigation is required to determine its impact on *LR* results.

### 4.4 The robustness of the model

The results of Experiment 1 showed that there was a significant liner correlation between $Log_{10}\left(LR\right)$ and *M_xn_*/*φ* among 17 tumor samples. When the reference was the true contributor, the model observed an increase in *LR* as the proportion of normal cells grew larger. This can be attributed to the gradual increase of the genotyping weight associated with normal cells. Conversely, in the non-contributor test, *LR* increased with increasing *M_xn_* as the weighting of the false contributor genotype decreased. Additionally, *LR* increased with increasing *φ* due to the sum of all genotype combination probabilities under *H_d_* becoming larger than the genotype probability under *H_p_*. The above results demonstrate the rationality of the model.

The efficacy of the model was evidenced by the results obtained from Experiment 2. *LR* calculation for 17 samples was much higher than 1, strongly supporting tumor samples derived from known individuals. Moreover, $Log_{10}\left(LR\right)$ remained linearly related to *M_xn_* for each sample. The *LR* appeared to decrease as the percentage of normal cells decreased. However, the correlation still needs to be verified with a large number of samples.

The 99.9% of the $Log_{10}\left(LR\right)$ was less than 0 for the non-contributor tests. Although there were some results that were slightly greater than zero, as observed when *M_xn_* reached its minimum value, this outcome is acceptable given the comparatively small *LR* of the corresponding sample for true contributor and the randomness of generating individual non-contributor. This manifests the specificity of the model to exclude irrelevant individuals through quantitative measurements.

Alleles of tumor cells are mutated from the somatic cell. The number of alleles shared with the latter should be greater than that of parent–offspring pairs or full-sibling pairs and equal to or slightly less than that of monozygotic twins (18). Relatedness was evaluated under *H_d_* to investigate whether individuals sharing some alleles with true contributors would be identified as contributors after model calculations. The result implies that the genotype probability is limited due to the additional information that the unknown individual is related to a specific known individual, which leads to the reduction of the *LR* while maintaining all values greater than 1. As a result, the *LR* value supports that the tumor tissue originated from a true contributor. In summary, the model rejects the hypothesis that the sample originates from individuals who are unrelated, parent–offspring pairs, or full-sibling pairs. Instead, it supports the hypothesis that the true contributor is the source of the sample.

Furthermore, the precision of the calculations was confirmed through the repetition of the analysis on both true contributors and non-contributors yielding consistent results (data not shown). The accuracy of the optimizer employed in this study was validated, and the consistent *LR* was obtained by comparing the outcomes of a two-person DNA mixture profile generated *in vitro* using our scripts and the Euroformix software. See **Supplementary Table 21** for details.

## 5 Conclusion

When comparing the STR profile of a tumor sample with a reference sample from the same individual, the genetic markers were no longer consistent. Using a probabilistic model to deal with the possibility of such alterations can provide a quantitative solution for their homology determination in clinical practice or forensic filed. In the present study, we attempt to provide a strength of evidence value that can be used for comparison and open up the prospect of using tumor samples for personal identification.

## Data availability statement

The datasets presented in this study can be found in online repositories. The names of the repository/repositories and accession number(s) can be found below: https://github.com/HYH-yuhan/TumorID, example.

## Ethics statement

The studies involving humans were approved by Ethics Review Board of Sichuan University. The studies were conducted in accordance with the local legislation and institutional requirements. The participants provided their written informed consent to participate in this study. Written informed consent was obtained from the individual(s) for the publication of any potentially identifiable images or data included in this article.

## Author contributions

YH: Conceptualization, Data curation, Formal analysis, Investigation, Methodology, Software, Validation, Visualization, Writing – original draft, Writing – review & editing. QZ: Conceptualization, Project administration, Supervision, Writing – review & editing. XD: Data curation, Methodology, Writing – review & editing. MZ: Resources, Supervision, Writing – review & editing. NC: Software, Validation, Writing – review & editing. HW: Software, Validation, Writing – review & editing. YTW: Conceptualization, Formal analysis, Writing – review & editing. YC: Conceptualization, Formal analysis, Writing – review & editing. YFW: Project administration, Resources, Supervision, Writing – review & editing. JZ: Funding acquisition, Project administration, Supervision, Writing – review & editing.
