# Supplementary material for: Exploration of identifying individual tumor tissue based on probabilistic model
Source: Front Oncol. 2024 Apr 23;14:1297135. doi: 10.3389/fonc.2024.1297135 (PMC11074449; doi:10.3389/fonc.2024.1297135)
Supplement: Supplementary file 1 [file DataSheet_1.docx]

Supplementary Material


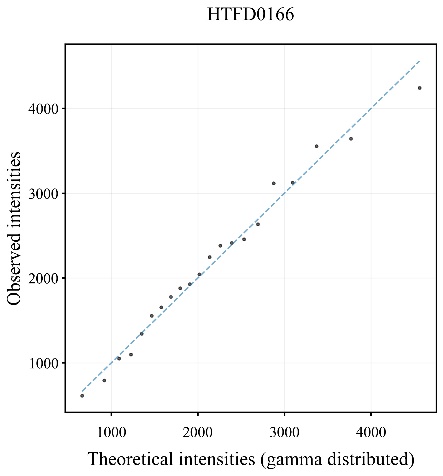

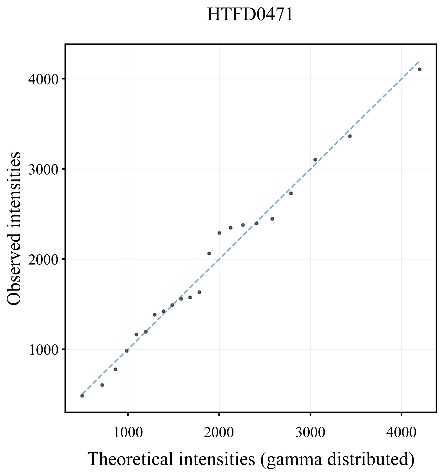

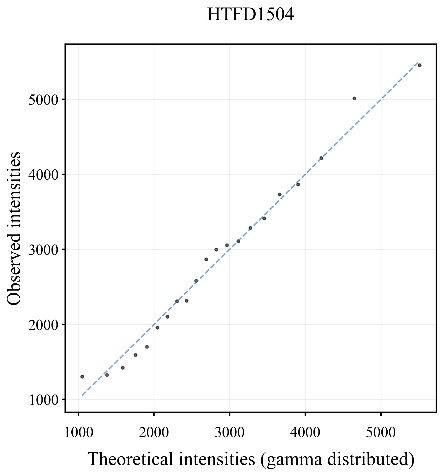

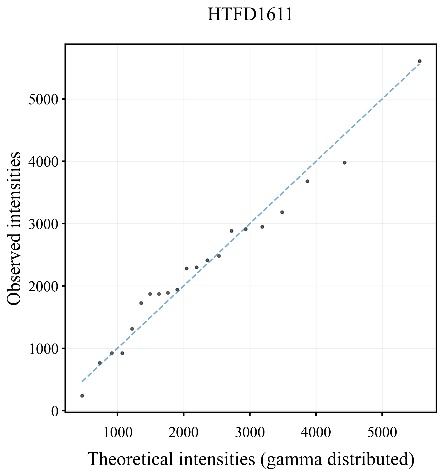

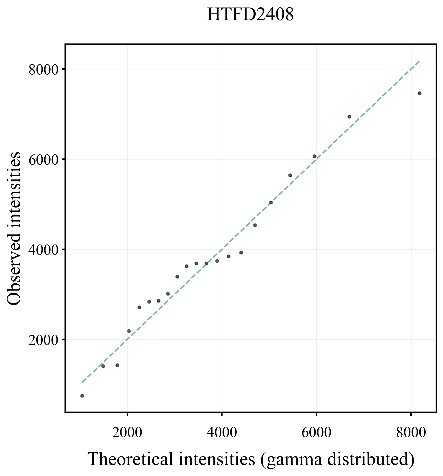

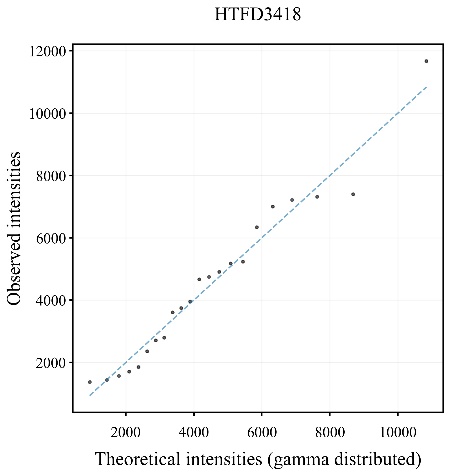

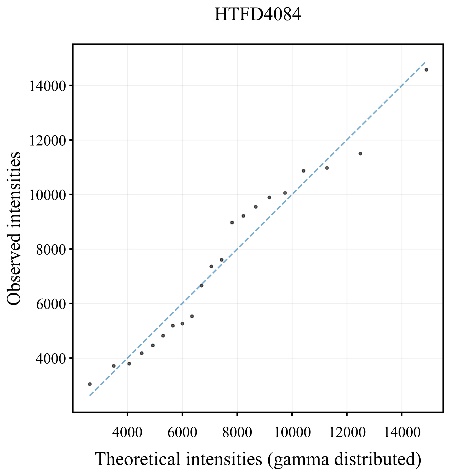

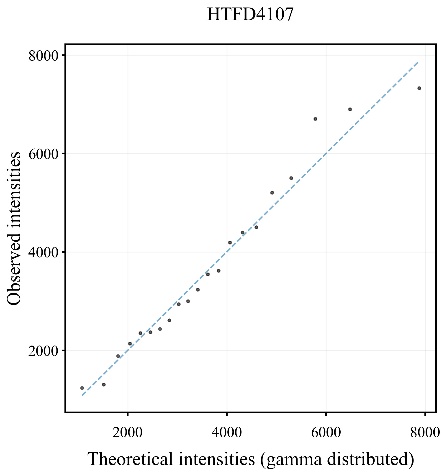

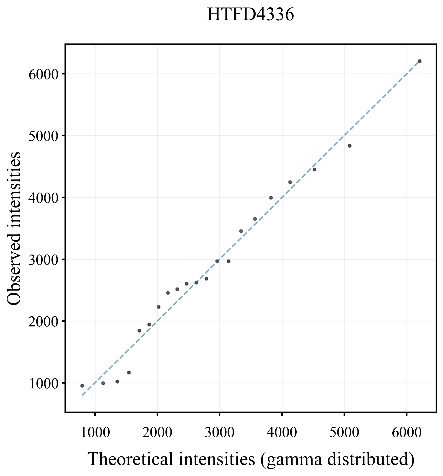

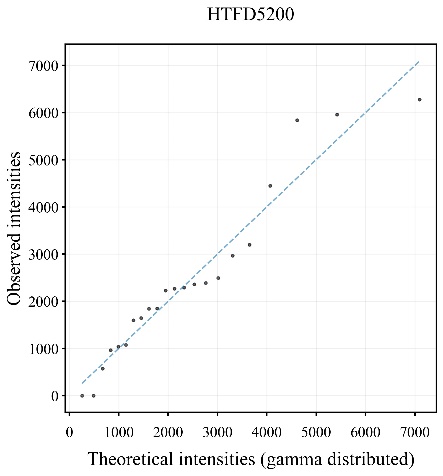

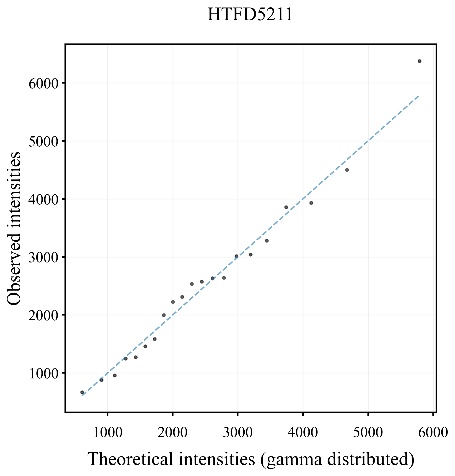

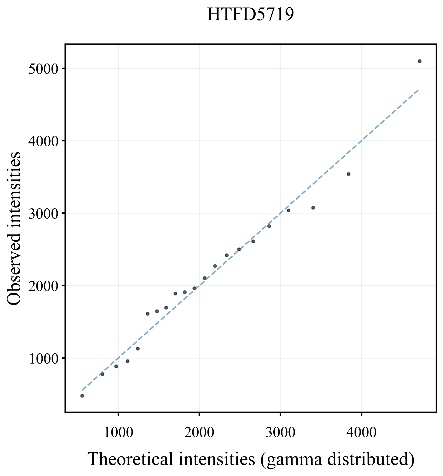

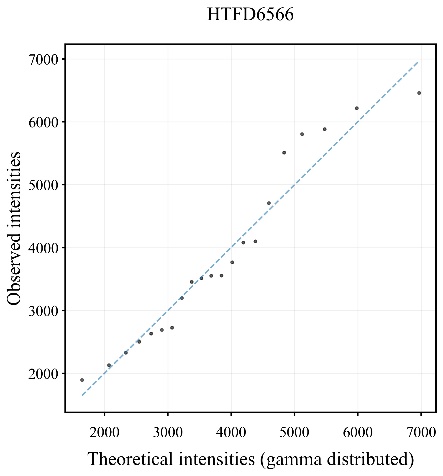

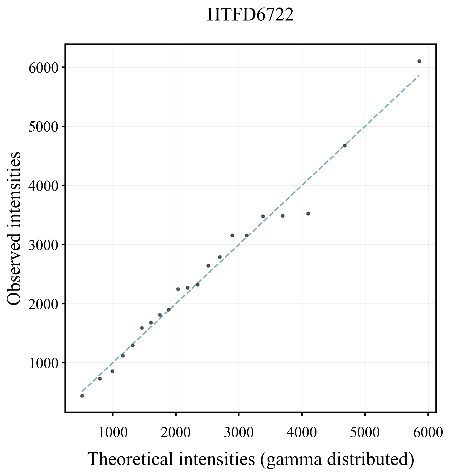

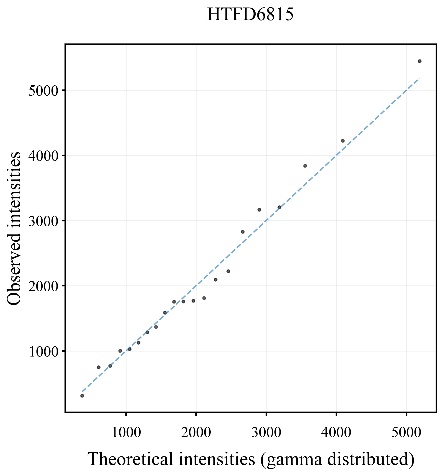

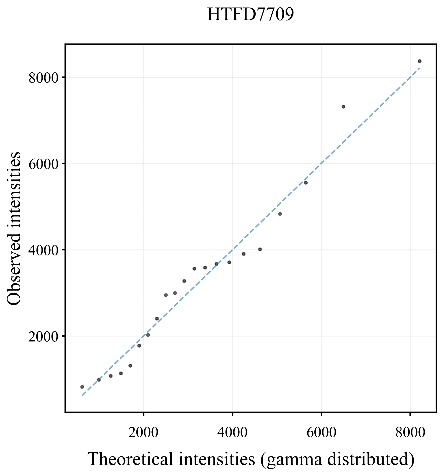

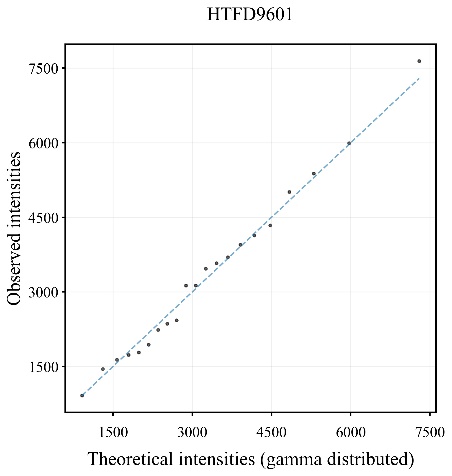


**Supplementary Figure 1.** Q-Q plot of peak height derived from DNA profile of all 17 samples. The fitted QQ plot’s horizontal coordinate is the percentile of gamma distribution (the parameter is derived from the maximum likelihood estimation of the peak height data of the profile), and the vertical coordinate is the percentile of the actual observation. All of them show that the scatter is basically distributed along the y=x line, indicating a satisfactory gamma fit.





































**Supplementary Figure 2.** The *LR* result of all 17 samples in experiment 1. $LR$ can be the smallest when $M_{x}(M_{xn})$ takes the minimum value and $\varphi$ takes the maximum value; $LR$ can be the largest when $M_{xn}$takes the maximum value and $\varphi$ takes the minimum value.





































**Supplementary Figure 3.** The *LR* result of all 17 samples in non-contributor test. The $LR$ value of the non-contributor test decreases with increasing $M_{x}(M_{xn})$ in each sample.
